# NanoDJ: a Dockerized Jupyter notebook for interactive Oxford Nanopore MinION sequence manipulation and genome assembly

**DOI:** 10.1186/s12859-019-2860-z

**Published:** 2019-05-09

**Authors:** Héctor Rodríguez-Pérez, Tamara Hernández-Beeftink, José M. Lorenzo-Salazar, José L. Roda-García, Carlos J. Pérez-González, Marcos Colebrook, Carlos Flores

**Affiliations:** 1 Research Unit, Hospital Universitario Nuestra Señora de Candelaria, Universidad de La Laguna, Santa Cruz de Tenerife, Spain; 2 Genomics Division, Instituto Tecnológico y de Energías Renovables (ITER), Santa Cruz de Tenerife, Spain; 3 Departamento de Ingeniería Informática y de Sistemas, Universidad de La Laguna, Santa Cruz de Tenerife, Spain; 4 Departamento de Matemáticas, Estadística e Investigación Operativa, Universidad de La Laguna, Santa Cruz de Tenerife, Spain; 5 CIBER de Enfermedades Respiratorias, Instituto de Salud Carlos III, Madrid, Spain

**Keywords:** Genome analysis, Nanopore sequencing, Jupyter, Docker

## Abstract

**Background:**

The Oxford Nanopore Technologies (ONT) MinION portable sequencer makes it possible to use cutting-edge genomic technologies in the field and the academic classroom.

**Results:**

We present NanoDJ, a Jupyter notebook integration of tools for simplified manipulation and assembly of DNA sequences produced by ONT devices. It integrates basecalling, read trimming and quality control, simulation and plotting routines with a variety of widely used aligners and assemblers, including procedures for hybrid assembly.

**Conclusions:**

With the use of Jupyter-facilitated access to self-explanatory contents of applications and the interactive visualization of results, as well as by its distribution into a Docker software container, NanoDJ is aimed to simplify and make more reproducible ONT DNA sequence analysis. The NanoDJ package code, documentation and installation instructions are freely available at https://github.com/genomicsITER/NanoDJ.

**Electronic supplementary material:**

The online version of this article (10.1186/s12859-019-2860-z) contains supplementary material, which is available to authorized users.

## Background

It has never been before so easy and affordable to access and utilize genetic variation of any organism and purpose. This has been motivated by the continuous development of high-throughput DNA sequencing technologies, most commonly known as Next Generation Sequencing (NGS). A key improvement is the possibility of obtaining long single molecule sequences with the fast and cost-efficiency technology released by Oxford Nanopore Technologies (ONT) and the marketing in 2014 of the MinION, a portable, pocket-size, nanopore-based NGS platform [1]. Since then, several algorithms and software tools have flourished specifically for ONT sequence data. Despite its size, it provides multi-kilobase reads with a throughput comparable to other benchtop sequencers in the market (1–10 Gbases by 2017), therefore still necessitating of efficient and integrated bioinformatics tools to facilitate the widespread use of the technology.

While MinION has shown promise in distinct applications [2], because of the low cost, laptop operability, and the USB-powered compact design of MinION, cutting-edge NGS technology is not any more necessarily linked to the established idea of a large machine with high cost that must be located in centralized sequencing centers or in a laboratory bench. As a consequence, the utility of MinION in field experiments to move from sample-to-answers on site have been demonstrated with infectious disease studies [3, 4], off-Earth genome sequencing [5], and species identification in extreme environments [6–8], among others. Leveraging of MinION capabilities in the academic classroom is a natural extension of these field studies to facilitate education of genomics in undergraduate and graduate students [9].

To date, there is no specific software solution aimed to facilitate ONT sequence analyses by integrating capabilities for data manipulation, sequence comparison and assembly in field experiments or for educational purposes to help facilitate learning of genomics [9]. We have developed NanoDJ, an interactive collection of Jupyter notebooks to integrate a variety of software, advanced computer code, and plain contextual explanations. In addition, NanoDJ is distributed as a Docker software container to simplify installation of dependencies and improve the reproducibility of results.

## Implementation

**Fig. 1:**
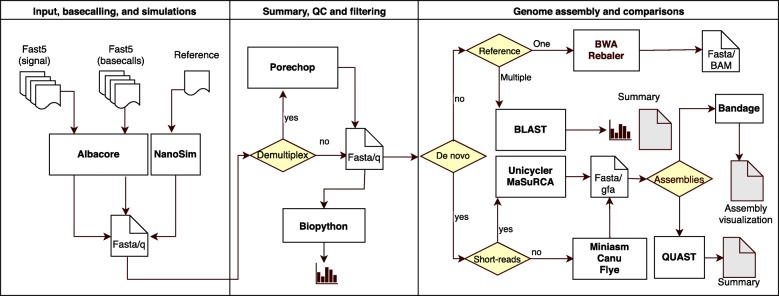
Simplified scheme of all NanoDJ functionalities

NanoDJ is distributed as a Docker container built underneath Jupyter notebooks, which is increasingly popular in life sciences to significantly facilitate the interactive exploration of data [10], and has been recently integrated in the widely used Galaxy portal [11]. The Docker container allows NanoDJ to run in an isolated, self-contained package, that can be executed seamlessly across a wide range of computing platforms [12], having a negligible impact on the execution performance [13]. NanoDJ integrates diverse applications (Additional file 1: Table S1) organized into 12 notebooks grouped on three sections (Fig. 1; Table 1). Main results are presented as embedded objects. In addition, one of the notebooks was conceived for educational purposes by setting a particularly simple problem and the inclusion of low-level explanations. To facilitate the use of the educational notebook and bypassing the installation of Docker and NanoDJ, a lightweight version of this notebook and small sets of ONT reads can be utilized from a web-browser using Binder (https://mybinder.org) in the NanoDJ GitHub repository. In addition, as part of the CyVerse project (https://www.cyverse.org/), NanoDJ has been incorporated into VICE, a visual and interactive computing environment that facilitates training of ONT data analysis. We illustrate the versatility of NanoDJ in distinct scenarios by providing results from four case studies (Additional file 1: Text S1).

**Table 1:** Summary of NanoDJ notebooks

Name	Functionality
0.0_QualityControl.ipynb	Evaluate the quality control and sequence handling
1.0_Basecalling.ipynb	Translates the events or the raw electrical signal from an ONT sequencer (FAST5 format) to a DNA sequence to obtain a FASTA or a FASTQ file
1.1_Trim+Demux.ipynb	Perform sequence trimming and demultiplexing
2.0_DeNovo_Canu-Miniasm.ipynb	De novo assembly with Canu or Miniasm, and polish with Racon and Pilon
3.0_DeNovo_Canu+polish.ipynb	Nanopolish modules to improve the Canu assembly
4.0_DeNovo_Flye.ipynb	De novo assembly with Flye software
5.0_DeNovo_Hybrid.ipynb	Perform de novo assembly of Nanopore reads in conjunction with Illumina reads using MaSuRCA and/or Unicycler software
6.0_AssemblyCompare.ipynb	Compare distinct assembly results based on QUAST software
7.0_SimulateReads.ipynb	Obtain simulated reads made with Nanosim software and the Nanosim-h fork with precomputed models
8.0_Alignment.ipynb	Reference-based assembly using either BWA, BLAST or Rebaler software
9.0_AssemblyGraph.ipynb	Assembly graph visualization
Educational.ipynb	Performs basecalling (with Albacore), quality control steps, and a BLAST-based classification of the reads (for educational purposes)

### Input, basecalling, and simulations

Input data can be a list of FAST5 files from previous basecalled runs (e.g. a Metrichor output) or event-level signal data to be basecalled using the latest ONT caller. The user can also simulate reads with NanoSim and pre-computed model parameters. This possibility is important in different scenarios as to help designing an experiment, or to bypass technical difficulties in academic setups [9].

### Summary, quality control and filtering

Either for a simulated or an empirical run, the user will obtain summary data and plots informing of read length distribution, GC content vs. length, and read length vs. quality score (when available). If barcodes were used in the experiment, Porechop can be used for demultiplexing, barcode trimming and to filter out reads.

### Genome assembly and comparison

Depending on the application, sequence data can be aligned against reference sequences or used for genome assembly using diverse methods. Alignment is performed either against one (BWA and Rebaler) or multiple (BLAST) reference sequences, providing the generation of BAM files for downstream applications (e.g., variant identification) or information of species composition. Alternatively, the user may opt for a de novo assembly. NanoDJ allows the use of some of the best-performing algorithms (Canu, Flye, and Miniasm), or to combine ONT reads with others obtained with second-generation NGS platforms for a hybrid assembly (Unicycler and MaSuRCA). The latter provides more effective assemblies and reduced error rate compared to assemblies based only on ONT reads [14]. NanoDJ includes the possibility of contig correction (Racon, Nanopolish, and Pilon). Assemblies can be evaluated with the embedded version of QUAST, and represented with Bandage.

## Limitations and future directions

For non-expert users, it would have been better if NanoDJ was envisaged as an on-line application to facilitate its use. However, our main objective was to integrate major tools for the analysis of ONT sequences in an interactive software environment to facilitate learning the basics behind ONT sequence analysis while providing a useful tool for professionals. Providing it as a Dockerized solution simply bolsters the focus on the use of the tool, reducing the burden of installing all dependencies by the user. At the moment, NanoDJ is set for the analysis of small genomes and targeted NGS studies, although focusing on primary and secondary analysis of DNA sequences. The integration of tools for variant identification and tertiary analysis (annotation of variants or sequence elements, interpretation, etc.) [15, 16], as well as for epigenetics [17] and direct RNA sequencing [18] will be the focus of further developments of NanoDJ.

## Conclusions

We present NanoDJ as an integrated Jupyter-based toolbox distributed as a Docker software container to facilitate ONT sequence analysis. NanoDJ is best suited for the analyses of small genomes and targeted NGS studies. We anticipate that the Jupyter notebook-based structure will simplify further developments in other applications.

## Availability and requirements

**Project name**: NanoDJ

**Project home page**: https://github.com/genomicsITER/NanoDJ

**Operating system(s):** Windows, Linux, Mac OS

**Programming language**: Bash/Python

**Other requirements**: Docker installation

**License:** GPL

**Any restrictions to use by non-academics**: None

## Additional file

Additional file 1: **Table S1.** Applications integrated in NanoDJ. **Text S1.** Testing on case study datasets. **Table S2.** Datasets for illustrative uses of NanoDJ. **Table S3.** Comparison of de novo assemblies using different inputs or with an assembly corrector. **Table S4.** Comparison of three de novo assemblers in a high-coverage ONT dataset. **Table S5.** Comparison of results from two hybrid de novo assemblers. **Figure S1.** Human mitochondrial DNA variant representation against the reference sequence. **Table S6.** Source of mitochondrial DNA genomes, simulations and classification results. (DOCX 1544 kb)
