# Supplementary material for: NanoDJ: a Dockerized Jupyter notebook for interactive Oxford Nanopore MinION sequence manipulation and genome assembly
Source: BMC Bioinformatics. 2019 May 9;20:234. doi: 10.1186/s12859-019-2860-z (PMC6509807; doi:10.1186/s12859-019-2860-z)
Supplement: Supplementary file 1 — Table S1. Applications integrated in NanoDJ. Text S1. Testing on case study datasets. Table S2. Datasets for illustrative uses of NanoDJ. Table S3. Comparison of de novo assemblies using different inputs or with an assembly corrector. Table S4. Comparison of three de novo assemblers in a high-coverage ONT dataset. Table S5. Comparison of results from two hybrid de novo assemblers. Figure S1. Human mitochondrial DNA variant representation against the reference sequence. Table S6. Source of mitochondrial DNA genomes, simulations and classification results. (DOCX 1544 kb) [file 12859_2019_2860_MOESM1_ESM.docx]

**Supplementary Material**

| **Table S1**. Applications integrated in NanoDJ. | | |
| --- | --- | --- |
| ***INPUT, BASECALLING AND SIMULATIONS*** | | |
| ***Application*** | ***Process*** | ***Result*** |
| Albacore^a^ v1.0.1 | Basecalling | FASTQ file |
| NanoSim-h^b^ v1.0.0.3 | Read simulation | FASTA file with simulated reads |
| ***SUMMARY, QUALITY CONTROL AND FILTERING*** | | |
| ***Application*** | ***Process*** | ***Result*** |
| Biopython^c*^ v1.70 | Summarization | Plots and tables |
| Porechop^d^ v0.2.2 | Demultiplexing, trimming and filtering | Demultiplexed FASTQ files |
| ***GENOME ASSEMBLY AND COMPARISONS*** | | |
| ***Application*** | ***Process*** | ***Result*** |
| BWA^e^ v0.7.17 | Alignment (one reference) | BAM file |
| Rebaler^f^ v0.1.0 | Alignment (one reference) | FASTA file |
| BLAST^g^ v2.7.1 | Alignment (multiple reference) | Read assignment (summary) |
| Miniasm^h^ *r104* | De novo assembly | Assembly files (FASTA, PAF) |
| Flye^i^ v2.3.1 | De novo assembly | Assembly files (FASTA) |
| Canu^j^ v1.6 | De novo assembly | Assembly files (FASTA, .gfa) |
| Racon^k^ v0.5.0 | Contig correction | Corrected FASTA |
| Nanopolish^l^ v0.8.5 | Contig correction | Corrected FASTA |
| Pilon^m^ v1.22 | Contig correction | Corrected FASTA |
| Unicycler^n**^ v0.4.1 | Hybrid de novo assembly | Assembly files (FASTA, .gfa) |
| MaSuRCA^o^ v3.2.2 | Hybrid de novo assembly | Assembly files (FASTA, .gfa) |
| QUAST^p^ v5.0.0 | Assembly comparison | Summary tables and plots |
| Bandage^q^ v0.8.1 | Visualization of assembly graphs (.gfa) | Assembly plot |
| ^a^https://nanoporetech.com/; ^b^Yang et al., 2017 (<https://pypi.python.org/pypi/NanoSim-H>); ^c^Cock et al., 2009 (www.biopython.org); ^d^https://github.com/rrwick/Porechop; ^e^Li & Durbin 2010 ([https://sourceforge.net/projects/bio-bwa/files](https://sourceforge.net/projects/bio-bwa/files/)); ^f^https://github.com/rrwick/Rebaler; ^g^Altschul et al., 1990 (<ftp://ftp.ncbi.nlm.nih.gov/blast/executables/blast+/LATEST>); ^h^Lin et al., 2016 (<https://github.com/lh3/miniasm>); ^i^Kolmogorov et al., 2018 (<https://github.com/fenderglass/Flye>); ^j^Koren et al., 2017 (<https://github.com/marbl/canu>); ^k^Vaser et al., 2017 (<https://github.com/isovic/racon>); ^l^https://github.com/jts/nanopolish; ^m^Walker et al., 2014 (<https://github.com/broadinstitute/pilon>); ^n^Wick et al., 2017 (<https://github.com/rrwick/Unicycler>); ^o^Zimin et al., 2013 ([ftp://ftp.genome.umd.edu/pub/MaSuRCA](ftp://ftp.genome.umd.edu/pub/MaSuRCA/)); ^p^Gurevich et al., 2013 (<https://sourceforge.net/projects/quast>); ^q^Wick et al., 2015 (https://rrwick.github.io/Bandage/)  *Dependencies included: Numpy, Matplotlib and Pandas.  **Dependencies included: Spades, Racon, Pilon, bowtie2, samtools, blast+. | | |

Supplementary Text 1. Testing on case study datasets

We illustrate the versatility of NanoDJ in distinct scenarios with four example datasets starting from diverse file inputs (Supplementary Table S2): (1) the assembly of a bacterial genome testing distinct *de novo* assemblers based on a high-coverage ONT data (different inputs); (2) a hybrid genome assembly of a bacterial genome based on low-pass ONT sequencing and short-read data at high coverage; (3) an emulation of a resequencing experiment to map ONT reads to a reference sequence for the identification of genetic variants (with third-party tools); and (4) an evaluation of species composition based on simulated ONT reads. All analyses were performed in a Ubuntu 16.04 Server with two Intel Xenon E5-2650 12-core 2.2 GHz processors and 256 Gb of RAM.

| **Table S2.** Datasets for illustrative uses of NanoDJ. | | | | | | | |
| --- | --- | --- | --- | --- | --- | --- | --- |
| Example | Organism | Size  (Mb) | Dataset | File(s) | Sequencing | #reads (x1000) | Source |
| 1 | *E. coli* | 4.6 | R9 | Fast5/Fasta | Rapid 1D | 164.47 | Web^a^ |
| 2 | *S. agalactiae* | 2.2 | R9/MiSeq | Fast5/Fastq | Rapid 1D/  PE300 | 3.72/2,975 | This study^b^ |
| 3 | *H. sapiens*  (mitochondria) | 0.016 | R9.4 | Fast5 | Rapid 1D | 59.36 | Web^c^ |
| 4 | Vertebrates  (mitochondria) | Variable | . | Fasta | Simulated | 0.21 | Web^d^ |
| ^a^http://lab.loman.net/2016/07/30/nanopore-r9-data-release  ^b^NCBI Sequence Read Archive IDs: PRJNA451111 (ONT), PRJNA451107 (Illumina)  ^c^https://github.com/nanopore-wgs-consortium/NA12878  ^d^http://hgdownload.soe.ucsc.edu/downloads.html  Mb: Megabases | | | | | | | |

**Example 1.** We used NanoDJ to test different *de novo* assemblers using default parameters in a 4.6 Mb reference *E. coli* genome (K-12-MG1655; NC_000913.3) obtained at a high coverage (>20X) with ONT. For these analyses we have used the following notebooks in the order: 1^st^) 1.0_Basecalling.ipynb, 2^nd^) 2.0_DeNovo_Canu-Miniasm.ipynb, 3^rd^) 4.0_DeNovo_Flye.ipynb, and 4^th^) 6.0_AssemblyCompare.ipynb. A first test with the most accurate assembler (Canu) compared the use of FAST5 files (333 Gb) and of a FASTA (1.5 Gb) file as the input dataset. In both cases, Canu assembled all reads into a single 4.6 Mb genome covering 99.9% of the reference. Using FASTA as the input did not reduced the elapsed time and introduced more mismatches and indels (Supplementary Table S3). The use of assembly polishers (e.g. Racon) did not improve significantly the assembly. Besides, we used the FASTA file to compare three distinct assemblers (Canu, Flye, and Miniasm). Overall, Canu and Flye were by far the most accurate alternatives, but Flye was much less computationally expensive. Miniasm was able to assembly the genome into a single contig, and was the fastest alternative of all three (Supplementary Table S4).

| **Table S3.** Comparison of Canu-based *de novo* assemblies of *E. coli* K-12-MG1655^$^ using different inputs or with an assembly corrector. | | | |
| --- | --- | --- | --- |
| Parameters | FASTA | FAST5 | FAST5-Racon |
| Total length assembled (bp) | 4,602,643 | 4,662,047 | 4,706,877 |
| Contigs (>=500 bp) | 1 | 1 | 1 |
| Genome fraction (%)* | 99.88 | 99.98 | 99.99 |
| Mismatches (#)* | 14,674 | 8,446 | 10,533 |
| Indels (#)* | 55,889 | 24,187 | 21,805 |
| N50 | 4,602,643 | 4,662,047 | 4,706,877 |
| GC (%) | 51.07 | 50.95 | 50.75 |
| Elapsed time (sec.) | 138,221.92 | 15,553.49 | 15,646.13 |
| ^$^Genome size and GC content are 4,641,652 bp and 50.79%, respectively.  *Against *E. coli* reference sequence NC_000913.3.  Indels: insertion/deletion variants; N50: minimum contig length to cover at least 50% of the genome; GC: guanine-cytosine content | | | |

| **Table S4.** Comparison of three de novo assemblers in a high-coverage ONT dataset (FASTA input) obtained from E. coli K-12-MG1655^$^. | | | |
| --- | --- | --- | --- |
| Parameters | Canu | Flye | Miniasm |
| Total length assembled (bp) | 4,602,643 | 4,678,264 | 4,404,394 |
| Contigs (>=500 bp) | 1 | 1 | 1 |
| Largest alignment (pb)* | 3,336,128 | 2,310,685 | 77 |
| Mismatches (#)* | 14,674 | 12,678 | 0 |
| Indels (#)* | 55,889 | 40,090 | 2 |
| N50 | 4,602,643 | 4,678,264 | 4,404,394 |
| GC (%) | 51.07 | 50.72 | 52.48 |
| Elapsed time (sec.) | 138,221.92 | 1,038 | 104 |
| ^$^Genome size and GC content are 4,641,652 bp and 50.79%, respectively.  *Against E. coli reference sequence NC_000913.3.  Indels: insertion/deletion variants; N50: minimum contig length to cover at least 50% of the genome; GC: guanine-cytosine content | | | |

**Example 2.** Here we used available *S. agalactiae* reads from a low-pass (~2.5X) ONT MinION experiment and 2,97 million reads (>300X) from a MiSeq (Illumina, Inc.) run to compare two state-of-the-art hybrid assemblers. In this case, we have used the following notebooks in the order: 1^st^) 1.0_Basecalling.ipynb, 2^nd^) 5.0_DeNovo_Hybrid.ipynb, 3^rd^) 6.0_AssemblyCompare.ipynb, and 4^th^) 9.0_AssemblyGraph.ipynb. NanoDJ integrated basecalling with Albacore, the hybrid assembly with Unicycler or MaSuRCA, and the assembly comparisons. Unicycler was superior to MaSuRCA based on the number of contigs, N50, the size of the largest contig, the number of mismatches and indels, and the proportion of covered genome (Supplementary Table S5). Plotting of Unicycler results also allowed isolating a 2.5 Kb plasmid sequence supported by our previous findings (unpublished), while MaSuRCA split its sequence into two small contigs.

| **Table S5.** Comparison of results from two hybrid *de novo* assemblers in a *S. agalactiae* dataset^$^. | | |
| --- | --- | --- |
| Parameters | Unicycler | MaSuRCA |
| Total length assembled (bp) | 2,159,288 | 2,099,176 |
| Contigs (>=500 bp) | 8 | 16 |
| Largest contig (bp) | 1,019,216 | 627,716 |
| Genome fraction (%)* | 98.57 | 95.29 |
| Largest alignment (pb)* | 1,018,789 | 627,116 |
| Mismatches (#)* | 165 | 230 |
| Indels (#)* | 38 | 53 |
| N50 | 714,293 | 434,380 |
| GC (%) | 35.39 | 35.47 |
| Elapsed time (sec.) | 9,836.52 | 3,009.03 |
| *^$^Streptococcus agalactiae* sequencing data was generated in house as part of an independent study using the Rapid Sequencing kit (SQK-RAD001) (Oxford Nanopore Technologies Ltd., Oxford, UK) in a single MinION 22-h run. Paired-end 300 bp reads for the same isolate were obtained using Nextera XT kit in a *MiSeq Reagent* kit V3 (Illumina, Inc., San Diego, CA), following the manufacturer's recommendations.  *Against *S. agalactiae* sequence NZ_CP010867.1.  Indels: insertion/deletion variants; N50: minimum contig length to cover at least 50% of the genome; GC: guanine-cytosine content | | |

**Example 3.** We used FAST5 files available for reads mapping to the human mitochondrial DNA (33 Gb) from the NA12878 human genome reference standard on the ONT MinION. NanoDJ was used to extract all reads and obtain a FASTQ file, integrate all reads into a single FASTA assembly by mapping against the revised Cambridge Reference Sequence (NC_012920, gi:251831106) with Rebaler, and obtain a BAM file, which was then manually inspected with IGV (Robinson et al., 2011) (Supplementary Fig. S1). In this case study, we have used the following notebooks in the order: 1^st^) 1.0_Basecalling.ipynb, 2^nd^) 0.0_QualityControl.ipynb, and 3^rd^) 8.0_Alignment.ipynb. The assembly was then used in HAPLOFIND, a third-party tool based on PhyloTree build 17 (Vianello et al., 2013), to confirm the classification of the reference data as H13a1a1 mitochondrial haplogroup.


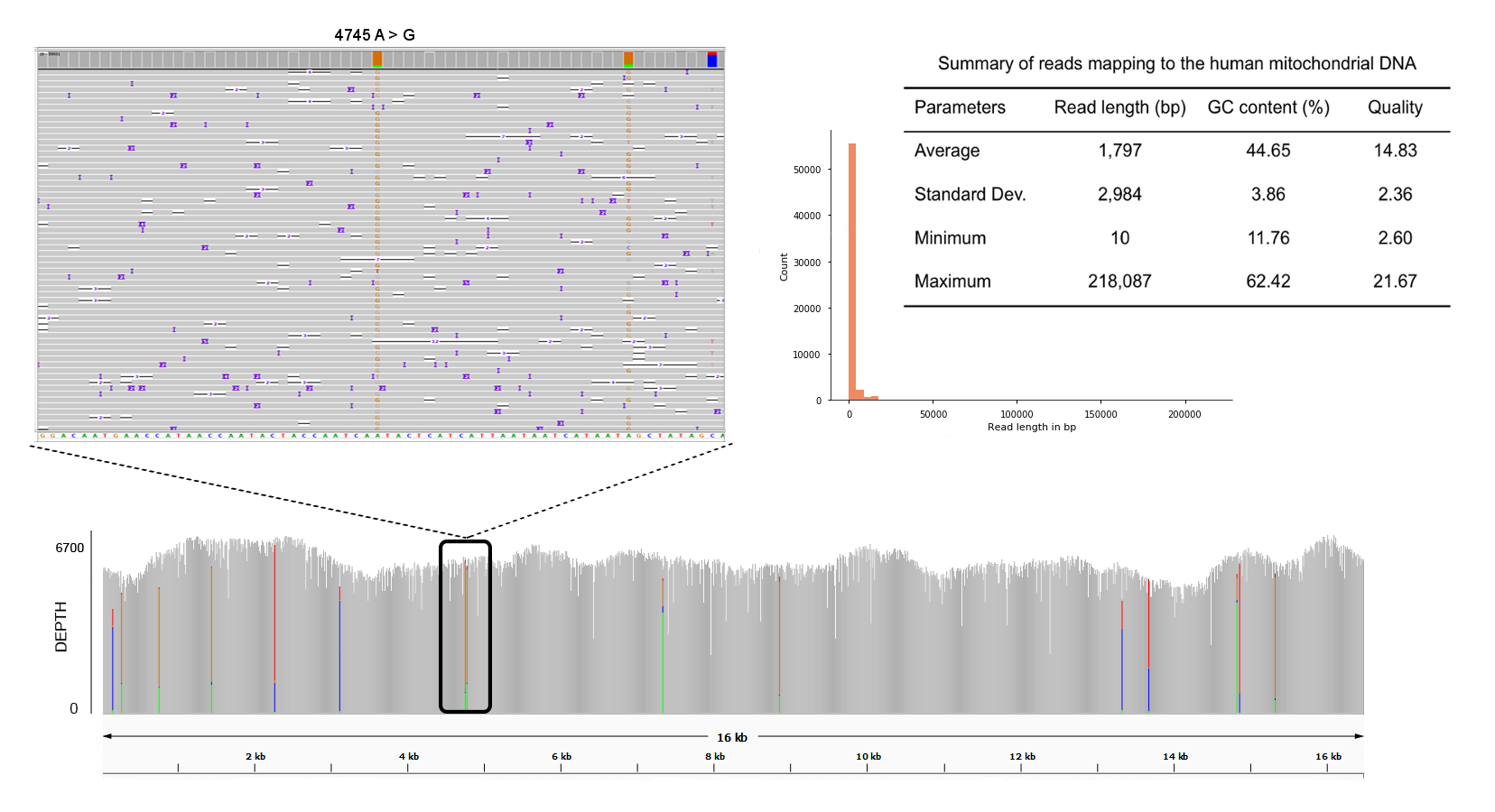


**Figure S1.** **Human mitochondrial DNA variant representation against the reference sequence (left), read distribution (right), and coverage information (bottom).** A consensus calling of variant alleles was made if the allele was present in >70% of reads. This identified the correct nucleotide at all reference positions, permitting the identification of the expected haplogroup for the reference DNA. The figure illustrates the case of position 4,745 where a A>G change is supported by the reads, defining H13a1 haplogroup.

**Example 4.** We used NanoDJ to create a local BLAST database with seven mitochondrial reference genomes from distinct vertebrates. In parallel, NanoDJ was used to simulate 30 ONT reads from seven species with NanoSim-h, which were merged into a single FASTA to emulate a 210-read heterogeneous sample run with balanced proportion of species (~14.3% each). NanoDJ was finally used for a BLAST-based classification of simulated reads to obtain species abundance. In this case study, we have used the following notebooks in the order: 1^st^) 7.0_SimulateReads.ipynb, 2^nd^) 0.0_QualityControl.ipynb, and 3^rd^) 8.0_Alignment.ipynb. As expected, the average abundance was supported by 14.3% of reads (excluding the unassigned), although with minimal fluctuations between 12.9 and 16.1% (Supplementary Table S6).

| **Table S6.** Source of mitochondrial DNA genomes, simulations and classification results. | | | | |
| --- | --- | --- | --- | --- |
| Species | Reference | Simulated reads | Assigned reads | Proportion* |
| *Gallus gallus* | Dec. 2015 (Gallus_gallus-5.0/[galGal5](http://genome.ucsc.edu/cgi-bin/hgGateway?db=galGal5)) | 30 | 27 | 14.5 |
| *Alligator mississippiensis* | Aug. 2012 (allMis0.2/[allMis1](http://genome.ucsc.edu/cgi-bin/hgGateway?db=allMis1)) | 30 | 25 | 13.4 |
| *Bos taurus* | Jun. 2014 (UMD_3.1.1/[bosTau8](http://genome.ucsc.edu/cgi-bin/hgGateway?db=bosTau8)) | 30 | 27 | 14.5 |
| *Equus caballus* | Sep. 2007 (Broad/[equCab2](http://genome.ucsc.edu/cgi-bin/hgGateway?db=equCab2)) | 30 | 29 | 15.6 |
| *Oreochromis niloticus* | Jan. 2011 (Nile tilapia/[oreNil2](http://genome.ucsc.edu/cgi-bin/hgGateway?db=oreNil2)) | 30 | 30 | 16.1 |
| *Rattus norvegicus* | Jul. 2014 (RGSC 6.0/[rn6](http://genome.ucsc.edu/cgi-bin/hgGateway?db=rn6)) | 30 | 24 | 12.9 |
| *Ovis aries* | Aug. 2012 (ISGC Oar_v3.1/[oviAri3](http://genome.ucsc.edu/cgi-bin/hgGateway?db=oviAri3)) | 30 | 24 | 12.9 |
| *Excluding 24 reads that were unassigned. | | | | |

**Supplementary References**

Altschul SF, Gish W, Miller W, Myers EW, Lipman DJ: **Basic local alignment search tool.** *J Mol Biol.* 1990, **215**:403–410.

Cock PJ, Antao T, Chang JT, Chapman BA, Cox CJ, Dalke A, Friedberg I, Hamelryck T, Kauff F, Wilczynski B, de Hoon MJ: **Biopython: freely available Python tools for computational molecular biology and bioinformatics.** *Bioinformatics*. 2009, **25**:1422–1423.

Gurevich A, Saveliev V, Vyahhi N, Tesler G: **QUAST: quality assessment tool for genome assemblies.** *Bioinformatics*. 2013, **29**:1072–1075.

Li: **Minimap and miniasm: fast mapping and de novo assembly for noisy long sequences.** *Bioinformatics*. 2016, **32**:2103–2110.

Li *&* Durbin: **Fast and accurate long-read alignment with Burrows-Wheeler Transform.** *Bioinformatics*. 2010, **26**:589–595.

Kolmogorov M, Yuan J, Lin Y, Pevzner P: **Assembly of long error-prone reads using repeat graphs.** 2018, https://doi.org/10.1101/247148.

Sergey Koren, Brian P. Walenz, Konstantin Berlin, Jason R. Miller, Adam M. Phillippy: **Canu: scalable and accurate long-read assembly via adaptive k-mer weighting and repeat separation.** *Genome Res.* 2016, **27**;722–736.

Robinson JT, Thorvaldsdóttir H, Winckler W, Guttman M, Lander ES, Getz G, Mesirov JP: **Integrative genomics viewer.** *Nat Biotechnol*. 2011, **29**:24–26.

Vaser R, Sović I, Nagarajan N, Šikić M: **Fast and accurate de novo genome assembly from long uncorrected reads.** *Genome Res.* 2017, **27**:737–746.

Vianello D, Sevini F, Castellani G, Lomartire L, Capri M, Franceschi C: **HAPLOFIND: a new method for high-throughput mtDNA haplogroup assignment.** *Hum Mutat.* 2013, **34**:1189–1194.

Walker BJ, Abeel T, Shea T, Priest M, Abouelliel A, Sakthikumar S, Cuomo CA, Zeng Q, Wortman J, Young SK, Earl AM: Pilon: **An integrated tool for comprehensive microbial variant detection and genome assembly improvement.** *PLoS ONE*. 2014, 9:e112963.

Wick RR, Judd LM, Gorrie CL, Holt KE: **Unicycler: Resolving bacterial genome assemblies from short and long sequencing reads.** *PLoS Comput Biol*. 2017, **13**:e1005595.

Wick RR, Schultz MB, Zobel J, Holt KE: **Bandage: interactive visualization of de novo genome assemblies.** *Bioinformatics*. 2015, **31**:3350–3352.

Yang C, Chu J, Warren RL, Birol I: **NanoSim: nanopore sequence read simulator based on statistical characterization.** *Gigascience*. 2017, 6:1-6.

Zimin AV, Marçais G, Puiu D, [Ro](https://www.ncbi.nlm.nih.gov/pubmed/?term=Roberts%20M%5BAuthor%5D&cauthor=true&cauthor_uid=23990416)berts M, Salzberg SL, Yorke JA: **The MaSuRCA genome assembler.** *Bioinformatics*. 2013, **29**:2669–2677.
